# Inter-Observer Agreement on Subjects' Race and Race-Informative Characteristics

**DOI:** 10.1371/journal.pone.0023986

**Published:** 2011-08-29

**Authors:** Heather J. H. Edgar, Shamsi Daneshvari, Edward F. Harris, Philip J. Kroth

**Affiliations:** 1 Department of Anthropology, University of New Mexico, Albuquerque, New Mexico, United States of America; 2 Maxwell Museum of Anthropology, University of New Mexico, Albuquerque, New Mexico, United States of America; 3 Health Sciences Library and Informatics Center, University of New Mexico, Albuquerque, New Mexico, United States of America; 4 Department of Orthodontics, University of Tennessee Health Sciences Center, Memphis, Tennessee, United States of America; The University of South Wales, Australia

## Abstract

Health and socioeconomic disparities tend to be experienced along racial and ethnic lines, but investigators are not sure how individuals are assigned to groups, or how consistent this process is. To address these issues, 1,919 orthodontic patient records were examined by at least two observers who estimated each individual's race and the characteristics that influenced each estimate. Agreement regarding race is high for African and European Americans, but not as high for Asian, Hispanic, and Native Americans. The indicator observers most often agreed upon as important in estimating group membership is name, especially for Asian and Hispanic Americans. The observers, who were almost all European American, most often agreed that skin color is an important indicator of race only when they also agreed the subject was European American. This suggests that in a diverse community, light skin color is associated with a particular group, while a range of darker shades can be associated with members of any other group. This research supports comparable studies showing that race estimations in medical records are likely reliable for African and European Americans, but are less so for other groups. Further, these results show that skin color is not consistently the primary indicator of an individual's race, but that other characteristics such as facial features add significant information.

## Introduction

Health and other socioeconomic disparities seem to be experienced along racial lines. However, contemporary research on race indicates that there is no biological validity to the concept [1]. Health disparities primarily result not from genetic variations associated with racial groups, but from socioeconomic factors that differentially affect individuals due to the racial group to which they are ascribed. For example, people with more stereotypical African skin color and facial features have been shown to receive poorer treatment than others (see [2] for a review; also see [3]). Data on race and ethnicity have long been collected in medical settings, and health disparities research has been based on these data. In order to determine reliability, a number of studies have compared two different sources of race and ethnicity data from medical and governmental sources. Some recent research has sought to go beyond basic categorization in medical records to examine correlations between race related phenotype (skin color) and health disparities [4], [5]. Understanding the limitations of any racial classification system is extremely important given that race is seen as a significant factor in making many governmental policy decisions regarding health care and other issues. Given that race is an extremely complex and dynamic concept, the quality of these policy decisions is limited by the inaccuracies inherent when racial data is not consistently and accurately acquired, maintained, and understood.

The research presented here addresses the question of reliability in two novel ways. First, the data derive from multiple observers who estimated a subject's race from the exact same information, orthodontic records that include subjects' names, addresses, and photographs. We used the current US census model for race estimation, allowing observers to choose one, many, all, or no race and ethnicity terms to describe the subject. We ask, when observers have the exact same information, do they reliably ascribe people to socially recognized groups? Second, in addition to having been asked to estimate subjects' races, observers were also asked to record which characteristics about a subject were informative about race. This allows us to examine to what degree skin color and other characteristics are informative to observers about a subject's race. Using this information we ask, how are ascriptions of race being made?

In addition to the applications to health and socioeconomic research, answering these questions can inform forensic research. For example, when people ascribe a missing person to a racial group, wide ranges of possible appearances are associated with that group. If there are some indicators of race that observers consistently agree are informative, investigators may be able to reliably predict the appearance of those features.

We use the word “race” rather than ancestry or ethnicity because it denotes a pattern of human variation that is the confluence of social groupings and biological characteristics, and best describes the groups studied here. While we consider categories the US Census denotes as both races (e.g., European or White Americans) and ethnicities (Hispanic or not Hispanic) we refer to all groups as races, as that best reflects the folk taxonomy of the area from which the data derive, Albuquerque, New Mexico, where 55.8% of Hispanics identify themselves as “some other race” [6]. Several researchers have suggested that this use of “some other race” implies identification with a Hispanic “race” [7], and our informal research agrees. Additionally, we use terminology to describe races that reflects a geographical area of ancestry (e.g., African American), unless referring to other authors' work, in which case we use the terminology used by those authors.

The University of New Mexico's Human Research Review Committee approved this research and a waiver of informed consent (protocol 05-410).

### Prior research on race agreement

This paper examines race agreement between two observers. This has been tested previously, but not necessarily with both observers having access to the exact same information or the same categorization scheme. Several studies have examined agreement between two sources of race and ethnicity data for a medical patient, such as birth and death records, or medical records and self-identification. In general, findings are that medical data sources are in good agreement for non-Hispanic European Americans and African Americans, and are less reliable for other groups, such as Asian Americans, Hispanic Americans, and Native Americans, e.g., [8]–[12]. Kressin et al. [11] examined agreement between two sources of data used by the Veteran's Administration. Among the 36% of their sample for which race and ethnicity were considered known, agreement was over 90% for African Americans and European Americans, between 65% and 85% for Asian Americans, Hispanic Americans, and Pacific Islanders. Agreement for Native Americans for the two sources was only 23%.

Maizlish and Herrera [13] compared patient self-identification as recorded in medical charts to data in an electronic patient management system used in several community clinics. Agreement between the two sources was 87% overall, but varied among clinics from 74% to 95%. The authors attributed the variation in agreement primarily to the use of different forms at the various clinics, so that the number of possible race and ethnicity labels available varied from 10 to 39.

Waldo [14] compared self-reported individual race and ethnicity statements originating in the Social Security Administration (SSA) and used by Medicare to self-reported race and ethnicity from the Medicare Current Beneficiary Survey (MCBS). As both data points were self-reports of group membership, one might expect disagreement to be extremely low. However, the two organizations collecting the data (SSA and MCBS) used different coding schemes. Results indicated that SSA coding was sensitive enough to provide accurate information on Whites and Blacks (accurate for over 95% of the instances where a respondent described himself as belonging to only one race). However, sensitivity for Asians, Native Americans, and Hispanics was 54.0%, 20.6%, and 35.7%, respectively. Therefore, any research conducted using data provided by the SSA would likely underestimate effects on these three groups.

In an interesting twist to understanding the relationship between race/ethnicity and health, Noymer et al. [15] compared race estimated by medical examiners to that described by the next of kin. They found that the odds of being classified as Native American were 2.9 times higher for people who died of cirrhosis, and the odds of being classified as African American on the death certificate were 2.4 times higher for homicide victims. The authors question the extent to which stereotypical thinking influences the data used in vital statistics.

### Prior research on indicators of race

Until recently, there has been little work examining possible relationships among disparities, skin color, and other characteristics such as facial features or hair form (but see [4], [5], [16]). However, such information could be used to better predict which individuals are most likely to experience disparities, providing a more nuanced understanding than race or skin color alone can provide. Colorism describes the situation of persons being treated differently according to their skin color, both within and across racial boundaries [17]. Variation in skin color has been shown to be related to several forms of discrimination in Hispanic Americans [18], Asian American women [19] and to affect attractiveness ratings in African Americans [20]. African Americans have higher blood pressure than other Americans [21], and within African Americans, darker skin color is associated with greater hypertension [22]. Additionally, stereotypical African facial features have been shown to be associated with negative judgments, even when separated from darker skin color [23] though how these negative judgments might be related to socioeconomic or health disparities are unknown.

## Materials and Methods

This study was conducted as part of a larger project to develop a web-based orthodontic case file system that is now freely available for research, education, and patient care (http://hsc.unm.edu/programs/ocfs). De-identified materials available on the Web include patient and treatment histories, diagnoses, demographics, intra-oral photographs, and x-rays. Identified data is secured in a restricted database and the physical collection including items such as full facial and profile photographs and patient names is housed at the University of New Mexico's Maxwell Museum of Anthropology . The identified data and physical collection are made available only to investigators with an IRB approved research protocol. The development of this database and case file system received approval from the University of New Mexico's Human Research Review Committee (protocol #05-410). In 2005, the University of New Mexico's Maxwell Museum of Anthropology acquired the James Economides Orthodontic Collection. The collection was compiled from 1972 through 1999, and consists of dental casts, cephalometric radiographs, photographs, and treatment records for 5,940 orthodontic patients, including records of approximately 600 sibling pairs and several multi-generational families. Approximately 400,000 photos/images and 20,000 x-ray films are included in the collection. These images represent the facial, skeletal, and dental variation of the contemporary Albuquerque population over the last 35 years, including people from a variety of ancestral backgrounds, including African, Asian, European, Hispanic, and Native American populations. The diverse population from which this sample is drawn is in many ways the database's greatest strength, but has also provided one of the project's greatest challenges. Patient records included neither the patients' estimations of their own ancestry (self identification) nor Dr. Economides' estimation (community or physician identification). However, since all the records are closed cases, each observer had access to the exact same information about each subject from which to form their estimation of race.

The process of coding for race is one that is extremely complex, dynamic, and not well standardized. We consulted the National Library of Medicine's Unified Medical Language System (UMLS) [24] to determine how many and which terms are used to reference race in biomedical research and practice. The UMLS is a metathesaurus, or thesaurus of thesauri, for the over 100 UMLS supported terminology or coding standards. The UMLS links the terms or codes from all these terminology standards by concept, so it is possible to determine what codes in what standards relate to the same concept. The complexity is related to the number of standards, the number of ways a given concept is represented or potentially represented in a number of different terminology standards, and the fact that terminology standards are periodically modified and updated over time. For example, the concept of “African American” is currently represented in 10 different biomedical terminology standards supported by UMLS. We found 500 unique concepts definitions that contain the text, “race” in the UMLS. Given there are 499 other UMLS-identified concepts related to “race,” the depth and breath of the complexity associated with coding for race in a standardized and coordinated manner is clear.

This project required the use of two coding systems, one for patient race, and one we developed *ad hoc* to code for the indicators observers used in making their estimates of patient race.

### Race classification

There are numerous terminology systems available for describing the variation present in U.S. populations. Most of these terminologies list groups under the overall rubrics of “race” and “ethnicity.” These categories are levels of socially ascribed folk taxonomies that often incorporate biological characteristics, such as skin color, as features used for group assignment [25]. Actual ancestry only overlaps with race and ethnicity to the extent that the biological characteristics used for group assignment are inherited [26]. Specificity of possible assignment varies among terminological systems. For example, the 2000 US Census listed five overall racial categories and two ethnicities [27]; the CDC currently lists nine overall racial categories with hundreds of subcategories that subsume ethnic coding [28], [29]. Even though there are significant ambiguities in identifying and classifying populations according to race and ethnicity, the allocation of public funds for various healthcare, education, and other public programs are frequently based on these classifications.

In order to determine what racial and ethnic categories should be included as variables in this database, while recognizing the ambiguity of racial and ethnic classification, we compared as many existing terminological schemes as possible to a set of three criteria:

1. Familiarity of coding to observers and projected database users
2. Use of coding in medical research
3. Specific applicability to the Economides Collection.

We determined to use a slightly modified version of the 2000 and 2010 US Census categories to code race for this database. While there are certainly weaknesses in the Census terminology, it was chosen because it is familiar to most Americans and is commonly used in medical research. The modification made reflects the specific population of Albuquerque and Dr. Economides' patient sample. New Mexico is 42.1% Hispanic, [6] a classification listed as an ethnicity in the Census' scheme. However, few people in New Mexico differentiate “Hispanic” as an ethnicity, and therefore include it as a different category with the other codes that are listed in the Census as races. Observers had the codes “African American,” “European American,” “Asian American,” “Hispanic American,” “Native American,” and “Native Hawaiian/Pacific Islander” available. Further, if they chose “Native American,” observers were asked whether they could estimate membership in one or more of the 22 Native American tribes present in New Mexico. Often, tribal affiliation could be determined from a patient's address (ex. “Zuni Pueblo”). This information was coded in the database using the tribal affiliation codes as defined in the National Register's list of federally recognized Tribes [30].

### Race indicators

While UMLS yielded many coding terminologies for racial and ethnic groups, it did not list any codes for race indicators. Three criteria were used to guide the development of race indicators observers could choose to mark as important in making their estimation of subjects' races:

1. Must be available in over 95% of records from materials such as intake forms, treatment records, and patient photographs
2. Must be informative about patients' biology or group affiliation
3. Must be available to an observer without special equipment or training, so that the parameters would be similar experienced by members of the general public.

Prior to the finalization of the database format, discussions were held with potential observers. Five indicators were determined to meet each of the three criteria:

1. Patient name
2. Patient address
3. Skin color
4. Facial features
5. Hair form and color.

Patient name, while not biological, often provides information about ancestry. For example, while “Smith” may not be informative about ancestry, “Chávez” is a common Hispanic last name in New Mexico, and “Begay” is common among Native Americans, especially Navajo. Patient address is included as a variable, but is only used to determine tribal affiliation as outlined above. Skin color, facial features, and hair form and color are available variables that indicate biological characteristics of patients. These characteristics are available to observers from the facial photos of each patient and demographic information contained in the physical collection.

Each characteristic was considered as a portion of the whole estimate. For example, while a last name may indicate a particular race or ethnicity, presence of such a name does not require membership in that group (Filipinos may have Spanish surnames but identify as Asian), and lack of an ethnic last name certainly does not preclude membership in a group (a subject could identify as Hispanic through his or her Mother's line, but have a surname associated with another group). The same uncertainty is true for any particular skin color, facial feature, or hair form. We provided for such situations with the flexibility afforded observers to choose one or more affiliations, associated with one or more characteristics.

### The classification process

There were eight different observers, and at least two observers examined each subject record. The observers were undergraduates, graduate students, or staff members in the Anthropology department at the University of New Mexico, ranging in age from 24–41. There were seven female observers. All but one of the observers had lived the majority of their lives in the western United States. Observers provided their own racial identification, according to the scheme described above. Seven observers self-identified as European American; one self-identified as African American and European American. Clearly, the observers do not represent a cross-section of the United States, nor even of Albuquerque. This lack of a representative sample of Americans as observers should be taken into account when considering results.

Each estimation of race was time stamped. Race estimations were based on subject names, full and profile facial photographs, intra-oral images, and address. Observers also knew the dates of the patient records. Observers were not provided any training or guidance in making race estimations, but were instead instructed to use their own life experience with the people they encountered around them. Any observer could choose not to estimate the race of any particular patient. However, in *post hoc* interviews, observers reported that they chose not to estimate race for a patient about 1% of the time. Additionally, only 2.7% of the patients in the database have only one race estimation, indicating refusal on an observer's part to estimate that patient's race. Given these low frequencies, observers' refusal to estimate race was not considered important. Finally, observers could choose any combination of single or multiple race estimations and indicators.

### Statistical analysis

Three statistical tests were applied to the inter-observer agreement data.

1. Analysis of variance (ANOVA) was used to test for significant differences among observers in their estimations of subjects' race and the indicators they selected as important in making their estimations [31].
2. The Kappa test (*K*) was used to statistically examine whether and to what degree agreement rates varied among the race categories [32], [33].
3. Odds ratios [34] were calculated to determine how agreement on the importance of indicators related to what might be expected by chance.

## Results

Observers made 4,226 observations of 1,919 subjects. Race estimations were made of each subject by at least two observers. Both observers only chose one race 3,042 times to describe 1,521 of these subjects. The category “Hawaiian/Pacific Islander” is not included in these results, as the category accounted for less than 0.2% of all choices. The pattern of results was the same for cases where both observers chose only one race (“one choice”) and cases where at least one observer chose more than one race (“all choice”), though in the latter case agreement rates were much weaker (overall 66%). The results described below are only for cases where both observers chose a single race to describe each subject. See the supporting information for results that include cases where at least one observer chose more than one race.

### Comparison of observers

For the cases where both observers chose only one race, observers made 3,042 observations. The average number of observations made is 379.5, but the range is from 16 to 1,096. Because some observers made many more observations than others, it was important to make sure that no single observer viewed subjects particularly differently than any other. Table 1 provides one choice frequencies and ANOVA results for two comparisons, the number of times each observer chose each race, and the number of times each observer chose each indicator. The P-value for the test of whether observers selected races significantly differently from each other is 1.00. For the test of whether observers chose race indicators significantly differently from each other, the P-value is 0.10. Therefore, disagreement about race or race indicators is equally distributed among the observers. Table S1 summarizes ANOVA tests for all choice comparisons observer agreement about race and race indicators.

**Table 1:** One choice analysis of variance testing for differences among observer's categorization of subjects' races and indicators of race.

		Race category					Indicator category				
Observer		African Am	Asian Am	European Am	Hispanic Am	Native Am	Address	Name	Skin color	Hair form	Facial features
1	n	5	6	82	82	5	2	154	148	64	161
	Freq	0.03	0.03	0.45	0.45	0.03	0.01	0.85	0.82	0.35	0.89
2	n	24	16	422	201	35	2	624	698	685	697
	Freq	0.03	0.02	0.60	0.29	0.05	0.00	0.89	1.00	0.98	1.00
3	n	40	22	723	263	48	9	177	1089	1084	1087
	Freq	0.04	0.02	0.66	0.24	0.04	0.01	0.16	0.99	0.99	0.99
4	n	6	10	146	78	7	0	169	245	238	240
	Freq	0.02	0.04	0.59	0.31	0.03	0.00	0.68	0.99	0.96	0.97
5	n	4	10	82	58	6	0	123	153	135	154
	Freq	0.02	0.06	0.51	0.36	0.04	0.00	0.76	0.95	0.84	0.96
6	n	1	2	201	11	6	1	22	218	109	109
	Freq	0.00	0.01	0.91	0.05	0.03	0.00	0.10	0.98	0.49	0.49
7	n	0	0	11	5	0	0	13	9	2	12
	Freq	0.00	0.00	0.65	0.29	0.00	0.00	0.76	0.53	0.12	0.71
8	n	17	11	222	154	14	0	306	404	194	386
	Freq	0.04	0.03	0.53	0.37	0.03	0.00	0.73	0.96	0.46	0.92
		ANOVA Sum of squares			F	Pr>F	ANOVA Sum of squares			F	Pr>F
Observer		0.0005			0.01	1.00	0.6086			1.95	0.10
Category		2.4089			71.41	<0.0001	4.1631			23.33	<0.0001

### Race agreement

**Figure 1:**
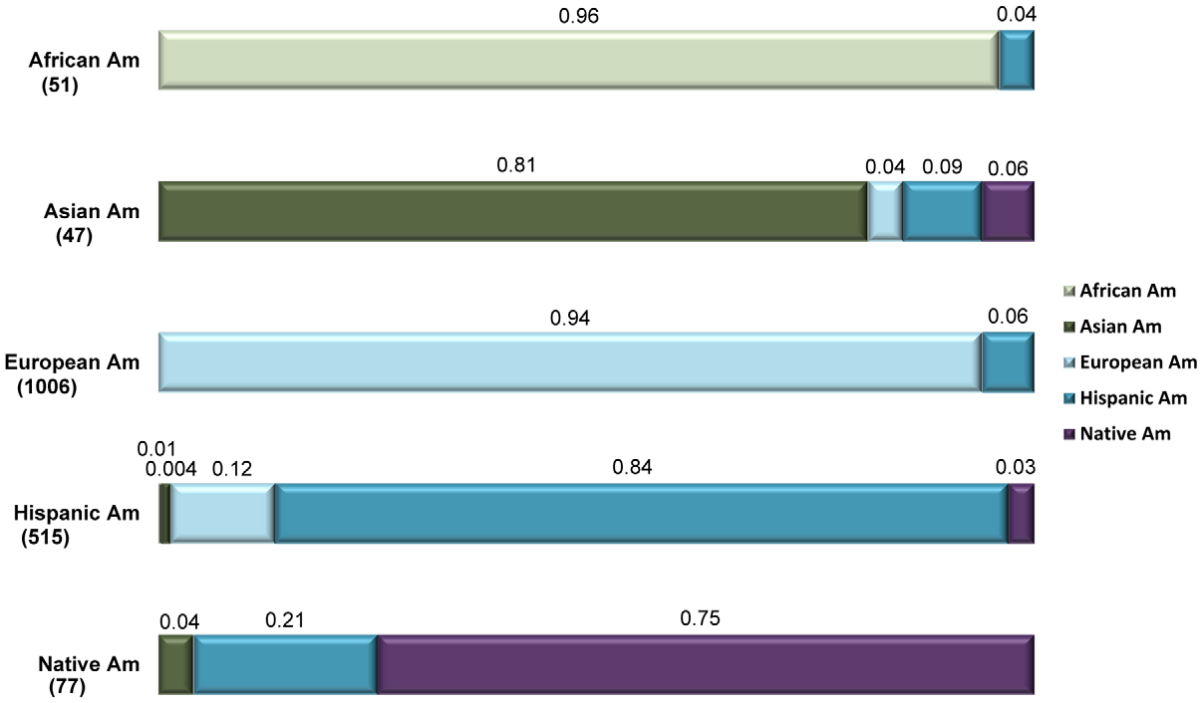
One choice inter-observer agreement and disagreement for race estimations. Bars represent one observer's estimates of race; colors represent the other's. Agreement is indicated where a bar and color represent the same race.

Figure 1 provides results of one choice inter-observer agreement and disagreement for race estimations. Each bar in the figure represents 100% of the times one observer estimated an individual belonged to a group. Each color represents the second observer's group estimations. Where the name of the bar and the group's color are the same, the two observers agreed in their estimate. Where the name of the bar is different than the group represent by a color, the two observers disagreed. For example, 94% of the time one observer estimated a subject to European American, the second observer also estimated that same individual as European American. In 6% of the cases where one observer estimated a subject to be European American, the second observer disagreed and estimated the subject to be Hispanic American.

As seen in previous studies [8]–[12] agreement is highest for African Americans (96.0%) and European Americans (94%). Agreement between two observers is lowest for Native Americans (75%), which is not the group that might have been predicted given previous research. Confusion between the two observers with regard to Native Americans is highest concerning Hispanic Americans. In 21% of the cases where one observer estimated a subject as Native American, the other observer chose Hispanic American. Additionally, in 12% of cases where one observer chose Hispanic American to describe a subject, the other observer chose European American. This ambiguity likely reflects the ancestry of New Mexican Hispanic Americans, who descend almost entirely from Native American and European/European American ancestors. Figure S1 provides all choice results of inter-observer agreement and disagreement, and can be interpreted in the same way as Figure 1.

A *K* of 1.0 indicates perfect agreement between two observers, while 0.0 indicates no greater agreement than would be expected by chance. *K* is high for all groups (see Table 2 for one choice results, Table S2 for all choice results), but much higher for African Americans (0.98) and European Americans (0.92) than for the other groups.

**Table 2:** One choice Kappa assessment and likelihood estimates of observer agreement on subjects' races.

	Concordance			
	Observed	Expected	*K*	Likelihood
African Am	48	1.64	0.98	45.16
Asian Am	38	1.17	0.89	45.02
European Am	943	599.66	0.92	176.8
Hispanic Am	431	138.47	0.87	258.27
Native Am	58	2.9	0.85	45.89

All likelihood estimates are significant at p<0.0001.

### Indicator agreement

Table 3 provides odds ratios for one choice inter-observer agreement, the case of both observers indicating that a characteristic is informative about a subject. The odds ratio is the odds of an event happening, divided by the probability that the event will not happen [35], [36]. An odds ratio above 1.0 signifies that the two observers both chose a characteristic more often than only one chose that characteristic, and that the indicator was informative about the subject. A number below 1.0 indicates that only one observer chose a characteristic more often than both observers chose it. This would suggest that the indicator is not reliably informative about a particular group.

**Table 3:** One choice odds ratios and [95% confidence intervals] for agreement between observers that race indicators are informative.

Indicator		Race (n)				
	All groups (1432)	African Am (48)	Asian Am (34)	European Am (914)	Hispanic Am (385)	Native Am (51)
Address	>999.99	<0.001	<0.001	<0.001	<0.001	>999.99
Name	4.37 [2.11–9.06]	<0.001	7.18 [3.23–15.99]	0.31 [0.24–0.39]	6.28 [4.82–8.18]	0.17 [0.06–0.47]
Skin color	1.75 [0.58–5.24]	0.43 [0.05–3.53]	0.17 [0.06–0.46]	3.77 [2.0–7.09]	0.78 [0.43–1.4]	0.12 [0.05–0.32]
Hair	1.57 [0.92–2.7]	2.97 [0.94–9.41]	3.1 [1.22–7.89]	1.33 [1.02–1.75]	0.66 [0.49–0.89]	0.76 [0.37–1.6]
Facial features	0.64 [0.29–1.39]	3.11 [0.35–27.8]	1.62 [0.32–8.12]	0.34 [0.22–0.53]	2.81 [1.68–4.71]	1.55 [0.56–4.32]

Table 3 indicates that the single most often agreed-upon indicator is name, driven by an odds ratio of 7.18 for Asian Americans and 6.28 for Hispanic Americans. However, name was absolutely not informative for African Americans (odds ratio <0.0001). Address information proved relevant only with regard to Native Americans, where it provided specific Tribal affiliation. Observers only selected it when they estimated a subject to be Native American. Since address is always associated with Native Americans, it is not informative about other groups.

For African Americans, the primary indicators of group affiliation are facial features (odds ratio 3.11) and hair form (odds ratio 2.97). Skin color is an unreliable indicator of African American affiliation, with an odds ratio that indicates observers disagree about its importance in estimating individuals' race (0.43). Skin color also is not informative for Asian Americans, Hispanic Americans, or Native Americans. Skin color is, however, important in estimating a person to be European American (odds ratio 3.77). See Table S3 for all choice odds ratio results.

## Discussion

The results described above are specific to Albuquerque, NM with the caveat that, while the subjects of the observations probably represent well the population of that city, the observers definitely do not. It is not known how generalizable our findings are to other cities or regions, or to the US population as a whole.

Given the same information, observers are relatively good at agreeing on the race of an individual, though this is less true for non-African or European Americans. Observers also generally agree on what characteristics are informative about a subject's racial affiliation. However, which characteristics are most informative varies among races. Names appear to be common indicators of Hispanic or Asian group membership. This is true despite that many individuals would be left out of these groups if names were to be the only indicator used. Additionally, skin color, traditionally considered a marker of membership in darker toned groups such as African Americans, instead is in this case to most clearly be indicating European American group membership. This phenomenon may specifically reflect the diverse population of New Mexico, where European Americans may be seen as having lighter skin, while all other groups are various darker shades that are mutually indistinguishable.

If we are not recognizing group membership efficiently, our understanding of how various segments of the population are affected by specific diseases may be skewed. For example, Clegg et al. [37] compared race and ethnicity coding in the Surveillance, Epidemiology, and End Results (SEER) program of the National Cancer Institute, which mainly comes from hospital records, to self-reported race and ethnic affiliation in the SEER National Longitudinal Mortality Study linked database. Overall agreement on race was high (K = 0.90). Agreement on ethnicity (Hispanic or not Hispanic) was much lower (K = 0.61). The authors conclude that in general cancer patients are under-recognized as minorities. Stewart and colleagues [38] looked specifically at agreement in ethnicity (Hispanic or non-Hispanic White) among cancer patients in California. The authors found that use of medical records alone or coupled with surname identification underestimated the number of Hispanics with cancer, as compared to self-identification.

In addition understanding how race and ethnicity are related to the epidemiology of specific diseases, classification of these groups may affect the data developed in clinical trials. At least since the 1993 National Institutes of Health revitalization act, funding agencies and researchers have made specific efforts to include minority groups in clinical research [39], [40]. The value of such efforts is potentially compromised by incorrect identification of research subjects.

Racial and ethnic classification is to some extent a moving target, in that how a person is classified today can be different from how he or she may have been classified 50 years ago, or could be classified 50 years from today [41]. In addition, it may be possible for the same observer to change classification decisions over time, as his or her exposure to persons of different groups changes. Observers are affected by their own racial and ethnic backgrounds, upbringing, and biases, as is evidenced by the imperfect agreement among multiple classifiers as well as individual's self-identification [11].

Recognizing the problems and ambiguities with classifying subjects by race and ethnicity, we realized that attempting to choose the perfect racial and ethnic coding system cannot solve this problem. Rather, we chose to design the database to have the capability to capture multiple racial and ethnic estimations by numerous observers, at multiple points in time, and record the associated characteristics on which observers based their estimations. At least two observers made independent estimations of race for each subject in the database. Our experience with using two observers during the data entry and verification process showed a reasonable agreement at a given point in time. Observers' self-identified ancestry in included in the database, as well as the date each classification was performed. This design will allow future users of the database to create queries to accommodate the ambiguities and limitations of racial and ethnic classification of the database as these categories change over time. This also provides anthropologists and other researchers with a powerful tool for studying racial perceptions in contemporary human societies.

There are at least two potential applications for this effort. First, users of the specific database for which this coding system was developed may opt to limit their studies to patients for whom all observers are in agreement, or alternatively, explore characteristics of individuals about which observers disagreed. Second, indicators of group membership, coupled with race agreement data, provide a new avenue of research into the nature of the taxonomy of race and ethnicity in the United States. Potential questions to be addressed with these data include why there seems to be higher agreement for some estimated races than others, and whether in other situations observers who agree on patients' races are using the same indicators to make their estimate.

While there is considerable research concerning recognition of individuals' faces both within and between racial groups [42] there is comparatively little work on how people recognize the racial affiliation of faces. Experimental evidence indicates that individuals' brains process faces of people of their own race differently than faces of people from another race [43]. However, social experience (exposure to different racial groups) may affect the way that people look at faces to estimate their race [44].

An interesting result concerns the lack of agreement among these observers that skin color is informative about group affiliation, beyond European American or not. This result is supported by early work in computerized facial color transfer [45]. By transferring actual skin tones among faces of people from various racial groups (African American, Asian American, European American) and testing facial recognition, these authors found that while skin color affects face perception, it is not the primary factor in determining whether faces are recognized. Instead, facial features such as nose, lip, and eye shape, may dominate the way individuals perceive other people's faces [45]. Recent anthropometric studies have found patterned differences in nose and midfacial measurements in Blacks, Whites, Chinese, and Koreans [46], [47]. Skin color may be useful alone to indicate race if European American vs. non-European American is the appropriate level of study. However, this new work with facial color transfer and facial anthropometrics, together with the results presented here, indicates that social science and health researchers using skin color as the sole or primary indicator of race may be missing important information.

This work is also a reminder to government agencies and other health policy decision makers that epidemiologic data stratified by race uses an imperfect, surrogate marker for various forms of the unequal distribution of health resources and socioeconomic opportunity. No matter how race is observed, it will always be a flawed proxy for the outcomes of complex, ever-shifting social forces.

## Supporting Information

Figure S1 **All choice results of inter-observer agreement and disagreement Bars represent one observer's estimates of race; colors represent the other's.** Agreement is indicated where a bar and color represent the same race.
(TIFF)

Table S1 **Summary of all choice ANOVA tests.**
(DOCX)

Table S2 **All choice Kappa assessment and likelihood estimates of observer agreement on subjects' races.** All likelihood estimates are significant at p<0.0001.
(DOCX)

Table S3 **All choice odds ratios and [95% confidence intervals] for agreement between observers that race indicators are informative.**
(DOCX)
