# Supplementary figures and images for: Inter-Observer Agreement on Subjects' Race and Race-Informative Characteristics
Source: PLoS One. 2011 Aug 29;6(8):e23986. doi: 10.1371/journal.pone.0023986 (PMC3163683; doi:10.1371/journal.pone.0023986)

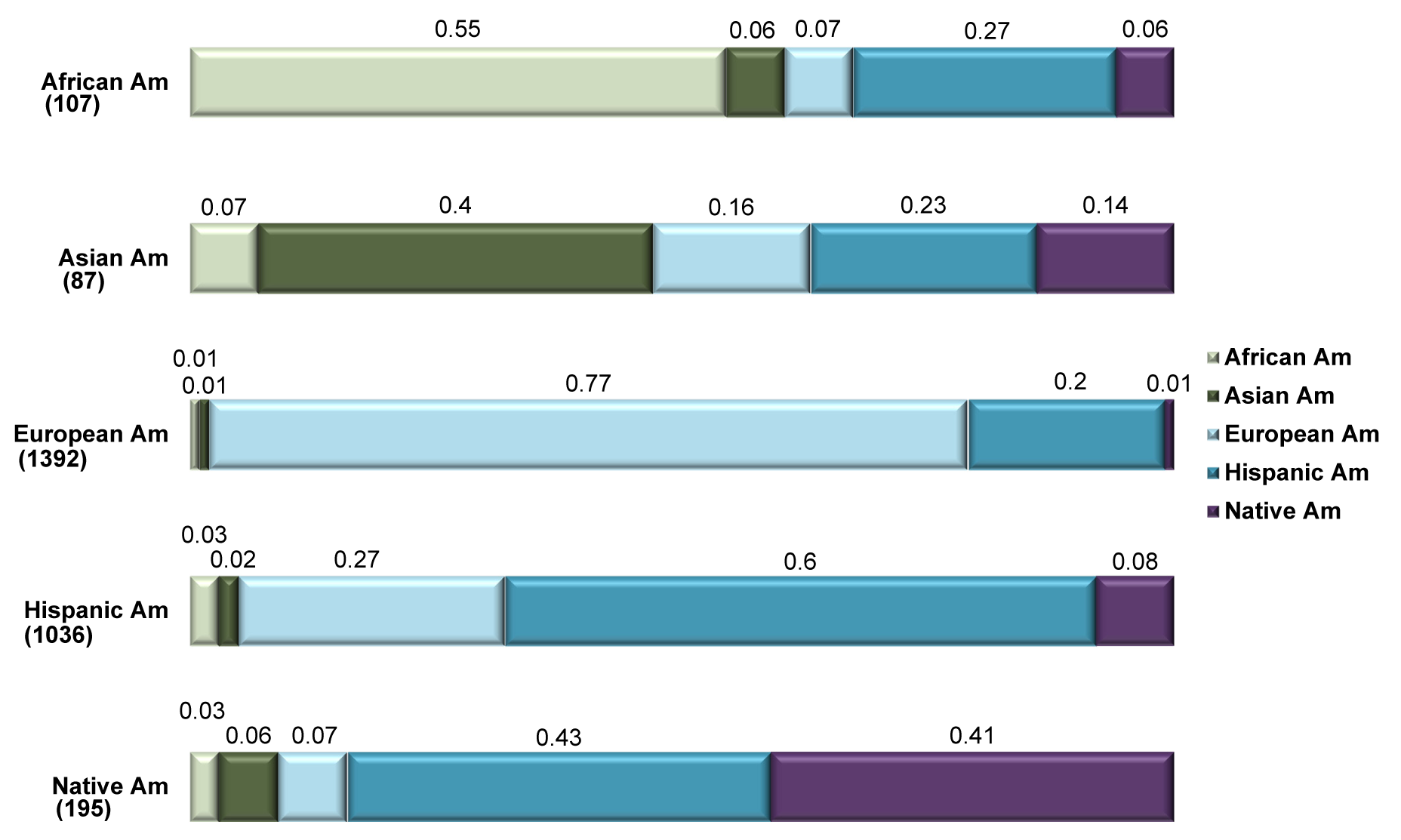

Supplement: Figure S1 — All choice results of inter-observer agreement and disagreement Bars represent one observer's estimates of race; colors represent the other's. Agreement is indicated where a bar and color represent the same race. (TIFF) [file pone.0023986.s001.tiff]
