# Supplementary material for: Inter-Observer Agreement on Subjects' Race and Race-Informative Characteristics
Source: PLoS One. 2011 Aug 29;6(8):e23986. doi: 10.1371/journal.pone.0023986 (PMC3163683; doi:10.1371/journal.pone.0023986)
Supplement: Table S1 — Summary of all choice ANOVA tests. (DOCX) [file pone.0023986.s002.docx]

| Test | ANOVA tests | Significant ANOVAS |
| --- | --- | --- |
| Did any observer choose any race significantly differently than any other? | 56 | 0 |
| Did any observer choose any race indicator significantly differently than any other? | 56 | 0 |
| Was any race or race indicator associated with any other race or race indicator significantly differently than others? | 110 | 51 |
|  | | |
