# Supplementary material for: Inter-Observer Agreement on Subjects' Race and Race-Informative Characteristics
Source: PLoS One. 2011 Aug 29;6(8):e23986. doi: 10.1371/journal.pone.0023986 (PMC3163683; doi:10.1371/journal.pone.0023986)
Supplement: Table S2 — All choice Kappa assessment and likelihood estimates of observer agreement on subjects' races. All likelihood estimates are significant at p<0.0001. (DOCX) [file pone.0023986.s003.docx]

|  | Concordance | | |  |
| --- | --- | --- | --- | --- |
|  | Observed | Expected | *K* | Likelihood |
| African Am | 59 | 3.13 | 0.71 | 61.72 |
| Asian Am | 35 | 1.48 | 0.58 | 30.73 |
| European Am | 1132 | 730.15 | 0.71 | 152.95 |
| Hispanic Am | 630 | 282.1 | 0.62 | 325.77 |
| Native Am | 79 | 7.58 | 5.5 | 46 |
