# Supplementary material for: Inter-Observer Agreement on Subjects' Race and Race-Informative Characteristics
Source: PLoS One. 2011 Aug 29;6(8):e23986. doi: 10.1371/journal.pone.0023986 (PMC3163683; doi:10.1371/journal.pone.0023986)
Supplement: Table S3 — All choice odds ratios and [95% confidence intervals] for agreement between observers that race indicators are informative. (DOCX) [file pone.0023986.s004.docx]

| Indicator |  | Race (n) | | | | |
| --- | --- | --- | --- | --- | --- | --- |
|  | All groups (1519) | African Am (49) | Asian Am (38) | European Am (943) | Hispanic Am (431) | Native Am (58) |
| Address | >999.99 | <0.001 | <0.001 | <0.001 | <0.001 | >999.99 |
| Name | 4.37 [2.11-9.06] | <0.001 | 7.18 [3.23-15.99] | 0.31 [0.24-0.39] | 6.28 [4.82-8.18] | 0.17 [0.06-0.47] |
| Skin color | 1.75 [0.58-5.24] | 0.43 [0.05-3.53] | 0.17 [0.06-0.46] | 3.77 [2.0-7.09] | 0.78 [0.43-1.4] | 0.12 [0.05-0.32] |
| Hair | 1.57 [0.92-2.7] | 2.97 [0.94-9.41] | 3.1 [1.22-7.89] | 1.33 [1.02-1.75] | 0.66 [0.49-0.89] | 0.76 [0.37-1.6] |
| Facial features | 0.64 [0.29-1.39] | 3.11 [0.35-27.8] | 1.62 [0.32-8.12] | 0.34 [0.22-0.53] | 2.81 [1.68-4.71] | 1.55 [0.56-4.32] |
|  | | | | | | |
